# Methotrexate-Induced Lymphoproliferative Disorder Complicating Into Spontaneous Tumor Lysis Syndrome and Disseminated Intravascular Coagulation

**DOI:** 10.7759/cureus.40665

**Published:** 2023-06-19

**Authors:** Abinash Parajuli, Justine Chinnappan, Qazi Azher, Ghassan Bachuwa, Philip J Mcdonald

**Affiliations:** 1 Internal Medicine, Hurley Medical Center/Michigan State University, Flint, USA; 2 Internal Medicine, Hurley Medical Center, Flint, USA; 3 Pathology and Laboratory Medicine, Hurley Medical Center, Flint, USA

**Keywords:** methotrexate-induced lymphoproliferative disorder, systemic lupus erythematosus, disseminated intravascular coagulation (dic), methotrexate, tumor lysis syndrome

## Abstract

Lymphoproliferative disorder (LPD) is a severe adverse outcome of methotrexate (MTX) administration in patients with rheumatoid arthritis (RA) and systemic lupus erythematosus (SLE). The immunosuppression caused is attributed to pathogenesis. Hence, discontinuation is the treatment. Reports on spontaneous tumor lysis with cessation of MTX are rare. We report a case of a female in her 50s with methotrexate-associated lymphoproliferative disease (MTX-LPD) following treatment for rheumatoid arthritis. Methotrexate was discontinued immediately. She presented two months later with severe disseminated intravascular coagulation (DIC) and spontaneous tumor lysis syndrome (STLS). Although tumor lysis syndrome responded well to rasburicase therapy, DIC was a challenge. MTX-LPD has various complications and highly variable presentation. RA/SLE patients receiving MTX should be regularly monitored, and MTX should be immediately stopped in suspicion of MTX-LPD. Although many patients respond to MTX cessation, some patients head to remission and relapse. At the same time, some worsen with complications such as DIC and tumor lysis syndrome, as described above. This case reiterates the need for regular monitoring following MTX therapy cessation for early identification and treatment of these complications to improve prognosis.

## Introduction

Methotrexate (MTX) is the first-line treatment for rheumatoid arthritis (RA) per the American College of Rheumatology (ACR) guidelines of 2021, both as monotherapy or in combination with other drugs, given the advantage of safety profile, low cost, and efficacy. Moreover, using MTX as monotherapy is recommended over hydroxychloroquine, sulfasalazine, or other disease-modifying agents of rheumatoid disease (DMARDs) for treatment-naive individuals with moderate to high inflammatory activity [1]. However, Anderson et al. reported a significantly increased occurrence of non-Hodgkin’s lymphoma in a cohort of American RA patients receiving methotrexate. At the same time, lymphoproliferative disorder (LPD) has also been reported in RA patients administered MTX [2]. After stopping MTX, 40%-50% of patients reported complete remission without additional medications [2]. However, the patient discussed here developed worsening lymphadenopathy and presented with spontaneous tumor lysis syndrome (STLS) and DIC even after the stoppage of MTX two months ago.

## Case presentation

**Figure 1:**
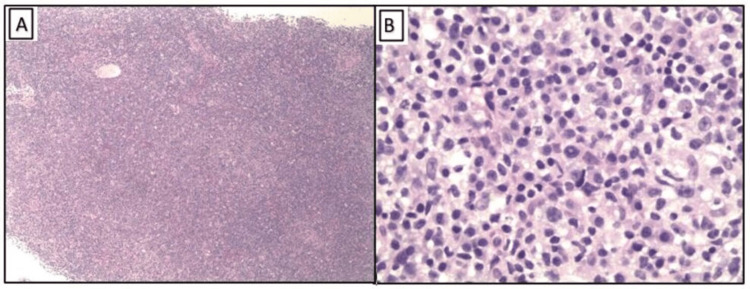
Hematoxylin and eosin stain of the lymph node showing complete effacement of the architecture (A, 4× magnification) with atypical lymphoplasmacytic infiltrate with lymphocytes of variable sizes (B, 40× magnification).

**Figure 2:**
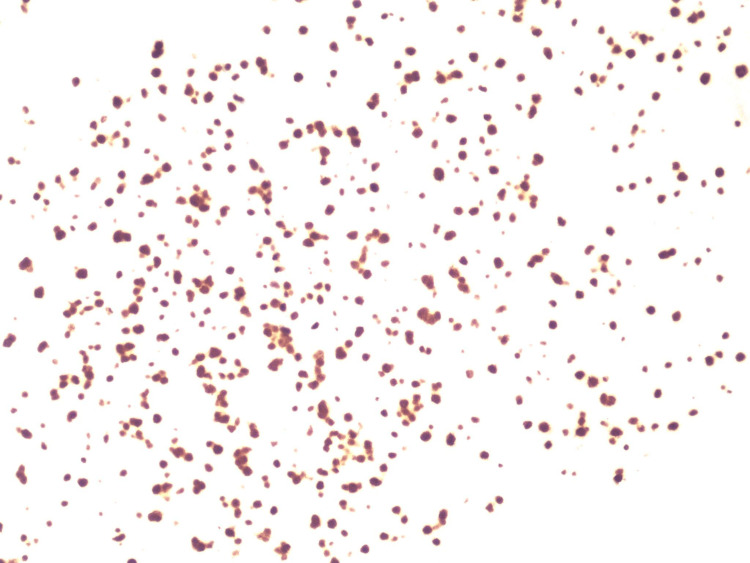
In situ hybridization for Epstein-Barr virus showing neoplastic cells positive for EBER (10× magnification). EBER: Epstein-Barr virus-encoded small RNA

**Figure 3:**
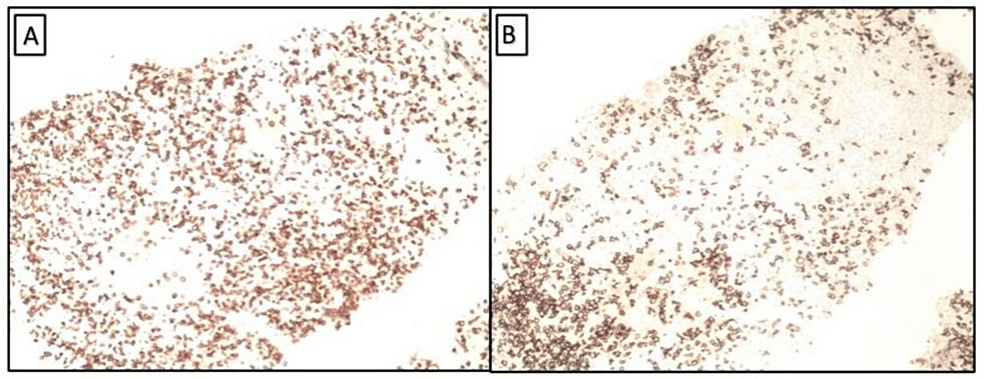
Immunohistochemical stain (10× magnification) with CD3 (A) and CD20 (B) showing positive staining indicating a mixture of T and B cells, respectively.

A 57-year-old female presented with nausea and vomiting, abdominal pain, dyspnea, and cough two months ago. Her comorbidities include paroxysmal atrial fibrillation on warfarin, rheumatoid arthritis(RA), and hypothyroidism. She was initially treated with hydroxychloroquine for RA, which was switched to methotrexate (MTX) for the past six months due to ophthalmic adverse effects. Two months ago, computed tomography (CT) revealed diffuse lymphadenopathy involving cervical, thoracic, and abdominal lymph nodes (LNs). The biopsy of the left iliac LN showed Epstein-Barr virus (EBV)-positive iatrogenic immunodeficient methotrexate-associated lymphoproliferative disorder, polymorphic type (Figures 1-3). The neoplastic cells were Epstein-Barr virus-encoded small RNA (EBER)-positive by in situ hybridization. Immunohistochemical stain showed lymphocytes, a mixture of CD20-positive B cells, CD3-positive T cells, and MUM1-positive plasma cells. Methotrexate was discontinued, and she was advised to follow up with the oncologist. She currently presented to the emergency department with epigastric pain, epistaxis, and dyspnea. On presentation, she was afebrile and hypotensive at 90/59 mmHg. On examination, the abdomen was diffusely tender without guarding or rebound tenderness. Laboratory findings on blood/serum are given in Table 1.

**Table 1:** Results of the serum/blood tests done at the time of presentation. WBCs: white blood cells, PT: prothrombin time, INR: international normalized ratio, aPTT: activated partial thromboplastin time

Laboratory test	Value	Reference range
WBCs	11 K/U	4-10.8 K/UL
Hemoglobin	7.7 g/dL	12-16 g/dL
Platelet	26 K/UL	130-430 K/UL
Peripheral smear	Schistocytes	-
Direct Coombs test	Positive	Negative
PT	49 seconds	12-14.7 seconds
INR	5.79	<4
aPTT	57 seconds	233.8-37.2 seconds
Fibrinogen	77 mg/dL	193-473 mg/dL
Lactate dehydrogenase	339 U/L	0-225 U/L
D-Dimer	3.37 mg/L	0-0.49 mg/L
Potassium	5.9 mEq/L	3.4-5.1 mEq/L
Phosphorus	4.7 mg/dL	2.7-4.5 mg/dL
Serum creatinine	1.7 mg/dL	0.5-1.1 mg/dL
Ionized calcium	1.37 mmol/L	1.10-1.30 mmol/L
Uric acid	14.6 mg/dL	2.6-7.2 mg/dL

**Figure 4:**
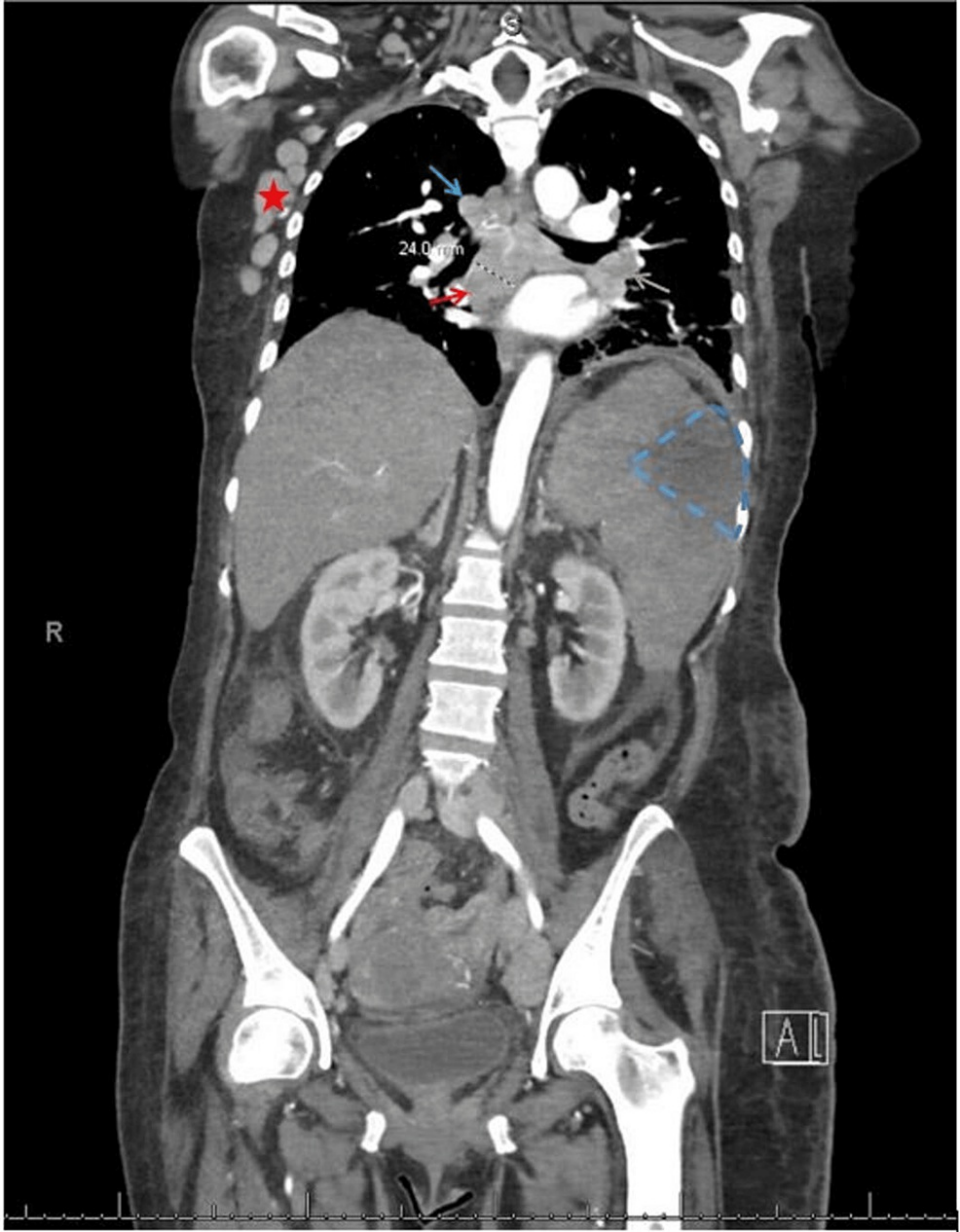
CT of the chest, abdomen, and pelvis (coronal view) showing multiple lymphadenopathies involving axillary (red star), hilar (green arrow), and mediastinal (blue arrow) groups with the largest being subcarinal lymph node (red arrow) measuring 2.4 cm in short axis. A wedge-shaped infarct is also seen in the upper portion of the spleen (blue dotted line). CT: computed tomography

Computed tomography (CT) of the abdomen and pelvis revealed a wedge-shaped splenic infarct and extensive lymphadenopathy involving the neck, axilla, mediastinum, retroperitoneum, pelvis, and inguinal region (Figure 4). CT of the head was unremarkable. Our patient met the laboratory criteria with elevated uric acid, phosphorus, and hypocalcemia. The presence of elevated serum creatinine and laboratory criteria led us to the clinical diagnosis of tumor lysis syndrome (TLS). Since this occurred without initiation of chemotherapy but with cessation of the MTX, it was considered spontaneous tumor lysis syndrome. She also had concomitant acute DIC due to underlying malignancy evident by thrombocytopenia, prolonged PT/INR, aPTT, decreased fibrinogen, and elevated D-dimer, and evidence of vascular thrombosis with splenic infarct. She also had positive direct Coombs with schistocytes in the peripheral smear, so a differential of autoimmune hemolytic anemia was also considered. A diagnosis of immune thrombocytopenic purpura (ITP) related to underlying connective tissue disease was also considered due to persistent platelet drop.

Given the diagnosis of TLS, she was started on aggressive hydration and rasburicase. Electrolytes, blood urea nitrogen (BUN)/creatinine (Cr), and uric acid showed improvement, and a second dose of rasburicase was given, after which uric acid came down to the normal level on day 6 of admission, at 6.1 mg/dL. Splenic infarct was managed conservatively. She was given fresh frozen plasma (FFP), cryoprecipitate, packed RBCs, and platelets for DIC. She was initially started on empiric antibiotics with cefepime and Flagyl for concerns of sepsis, which were discontinued after a week as no source of infection was identified. Prednisone was initiated as well for autoimmune hemolytic anemia. The oncologist also started her on Rituxan for ITP. Despite all the measures, her hemoglobin and platelets continued to drop. Hence, intravenous immunoglobulin (IVIG) 1 g/kg/day was tried for two days with a plan to taper steroids. The patient did not respond to the above therapy and required platelet and RBC transfusion on a daily basis. Taking into consideration her performance status and acuity of symptoms, she was not considered a candidate for multi-agent chemotherapy, which could be used to treat this type of lymphoma.

The patient started refusing treatment. Hence, goals of care were discussed with her and her family. She was discharged home under hospice care.

## Discussion

Iatrogenic immunodeficiency-associated lymphoproliferative disorders (OII-LPD) are defined as lymphoma or lymphoid proliferations arising in patients on immunosuppressive drugs for autoimmune disease. The unique nature of OII-LPD is the spontaneous resolution after cessation of therapy. Its course can be subclassified as regression (70%), including brief regression followed by either recurrence or relapse, and progression (30%). One-third of patients undergoing regression do so only transiently before having a relapse or recurrence [3,4]. In general, RA is associated with a higher risk for lymphoma, but there are only limited studies demonstrating the association of MTX use in RA and lymphoma [5]. Rheumatoid arthritis-associated lymphoproliferative disorders (RA-LPDs) were mostly associated with female predominance, younger age of onset, unfavorable prognosis, and EBV positivity compared to sporadic LPD [6]. Our patient was a female, diagnosed in her 30s, and the LPD was positive for EBV.

Tumor lysis syndrome (TLS) is an oncological emergency commonly associated with hematologic malignancies and seldom with solid tumors [7]. Per Cairo and Bishop’s definition, TLS is categorized as laboratory and clinical [8]. Laboratory TLS is defined by the presence of two or more metabolic abnormalities: hypocalcemia, hyperkalemia, hyperuricemia, and hyperphosphatemia. These abnormalities should be demonstrated from three days before initiation of therapy to seven days after commencement of the therapy. Clinical TLS is defined as meeting the laboratory criteria for TLS along with one of the following: increased creatinine level, seizures, cardiac dysrhythmia, or death [8]. Our patient met the criteria of both clinical and laboratory tumor lysis syndrome. The various modalities that govern the occurrence and severity of TLS include tumor burden, the characteristics of the patient (preexisting nephropathy, dehydration, hypotension, exposure to nephrotoxins, etc.), and supportive care (hydration) [7]. Although TLS classically occurs following any type of therapy for cancer, there have been rare reports of spontaneous TLS (STLS) [9]. Although it is rarer in solid tumors, its occurrence is found to be associated with poor prognosis and increased mortality in various studies [10]. The standard of treatment for TLS includes aggressive hydration and reducing the level of uric acid with the use of rasburicase. These measures help in restoring renal perfusion with the added benefit of decreasing the phosphorus level [11]. Rasburicase is considered superior to allopurinol in this condition as it is more effective in preventing xanthine accumulation and by directly breaking down uric acid [11]. Our patient responded well with aggressive IV hydration, and serum uric acid normalized after the second dose of rasburicase. There are no standard treatment guidelines for non-regression of LPD after methotrexate therapy cessation, but ABVD or the combination of brentuximab vedotin with AVD can be tried [12,13].

Disseminated intravascular coagulation (DIC) is characterized by extensive intravascular fibrin formation due to excessive blood protease activity that overcomes the natural anticoagulation mechanisms [14]. Several pathological conditions may trigger DIC, with trauma, cancer, sepsis, and pregnancy being the commonest [11]. The mortality and morbidity associated with DIC are primarily related to the underlying disease rather than the complications of DIC [14]. The control or elimination of the underlying cause should therefore be the primary concern [12]. Our patient with laboratory features consistent with DIC along with epistaxis was initially transfused with FFPs, cryoprecipitate, platelets, and packed RBCs. Fibrinogen showed some improvement, while platelet count continued to decline. ITP was suspected, in addition to DIC, as a cause of thrombocytopenia, but steroids did not help. Commencement of IVIG did ameliorate the thrombocytopenia, but with a poor prognosis and refusal of treatment by the patient after that, she was transitioned to hospice care. In conclusion, we discussed a patient with MTX-LPD presenting after two months of stopping MTX with spontaneous tumor lysis syndrome and DIC. Tumor lysis syndrome improved with aggressive hydration and rasburicase. Thrombocytopenia did not improve with platelet transfusion, steroid therapy, or IVIG. This case provides a different perspective that all patients do not go into remission after cessation of MTX. Some may present with severe complications. Patients diagnosed with MTX-LPD should be routinely monitored for any worsening symptoms, especially after discontinuing the immunosuppressive therapy, as the restoration of the immune system as such can act equivalent to chemotherapy-causing TLS.

To our knowledge, we are the first to report MTX-LPD complicated by spontaneous TLS and DIC. Further data should be gathered to determine the prognostic factors for complications of MTX-LPD, and standard guidelines need to be established in monitoring these patients to facilitate early diagnosis with larger studies.

## Conclusions

This case provides a different perspective that all patients do not go into remission after cessation of MTX. Some may present with severe complications. Patients diagnosed with MTX-LPD should be routinely monitored for any worsening symptoms, especially after discontinuing the immunosuppressive therapy, as the restoration of the immune system as such can act equivalent to chemotherapy-causing TLS. To our knowledge, we are the first to report MTX-LPD complicated by spontaneous TLS and DIC. Further data should be gathered to determine the prognostic factors for complications of MTX-LPD, and standard guidelines need to be established in monitoring these patients to facilitate early diagnosis with larger studies.
